# A “Crossomics” Study Analysing Variability of Different Components in Peripheral Blood of Healthy Caucasoid Individuals

**DOI:** 10.1371/journal.pone.0028761

**Published:** 2012-01-12

**Authors:** Kristina Gruden, Matjaž Hren, Ana Herman, Andrej Blejec, Tanja Albrecht, Joachim Selbig, Chris Bauer, Johannes Schuchardt, Michal Or-Guil, Klemen Zupančič, Urban Švajger, Borut Štabuc, Alojz Ihan, Andreja Nataša Kopitar, Maja Ravnikar, Miomir Knežević, Primož Rožman, Matjaž Jeras

**Affiliations:** 1 National Institute of Biology, Ljubljana, Slovenia; 2 Centre of Excellence for Biosensorics, Instrumentation and Process Control, Solkan, Slovenia; 3 Blood Transfusion Centre, Ljubljana, Slovenia; 4 University of Potsdam, Potsdam, Germany; 5 MicroDiscovery GmbH, Berlin, Germany; 6 Humboldt-University, Berlin, Germany; 7 Gastroenterological Clinic, University Medical Centre Ljubljana, Ljubljana, Slovenia; 8 Medical Faculty, University of Ljubljana, Ljubljana, Slovenia; 9 Celica Biomedical Centre, Ljubljana, Slovenia; 10 Faculty of Pharmacy, University of Ljubljana, Ljubljana, Slovenia; 11 Biobanka Ltd, Ljubljana, Slovenia; Governmental Technical Research Centre of Finland, Finland

## Abstract

**Background:**

Different immunotherapy approaches for the treatment of cancer and autoimmune diseases are being developed and tested in clinical studies worldwide. Their resulting complex experimental data should be properly evaluated, therefore reliable normal healthy control baseline values are indispensable.

**Methodology/Principal Findings:**

To assess intra- and inter-individual variability of various biomarkers, peripheral blood of 16 age and gender equilibrated healthy volunteers was sampled on 3 different days within a period of one month. Complex “crossomics” analyses of plasma metabolite profiles, antibody concentrations and lymphocyte subset counts as well as whole genome expression profiling in CD4^+^T and NK cells were performed. Some of the observed age, gender and BMI dependences are in agreement with the existing knowledge, like negative correlation between sex hormone levels and age or BMI related increase in lipids and soluble sugars. Thus we can assume that the distribution of all 39.743 analysed markers is well representing the normal Caucasoid population. All lymphocyte subsets, 20% of metabolites and less than 10% of genes, were identified as highly variable in our dataset.

**Conclusions/Significance:**

Our study shows that the intra-individual variability was at least two-fold lower compared to the inter-individual one at all investigated levels, showing the importance of personalised medicine approach from yet another perspective.

## Introduction

Advanced therapies of complex diseases such as cancer and autoimmune disorders are being evaluated in a number of clinical studies worldwide. The majority of clinical studies provide reports containing results exclusively related to the groups of patients involved, segregated according to different treatment protocols, the efficacies of which are being cross-evaluated and compared. In many cases historical data are considered for comparison while the healthy population control values are only used in a very limited extent or not applied at all [1], [2]. Especially when different immunological parameters are being evaluated such normal healthy controls are indispensible for proper interpretation of complex experimental datasets and should therefore be readily accessible to the research community.

Peripheral blood is the most easily accessible human tissue. Through analysis of its cellular components as well as numerous soluble factors, we can assess the (patho)physiological state of the organism. One of the most promising approaches for its evaluation are the so called “omics” technologies that enable holistic insight into studied system [3]. Until now several studies analysing separate “omes” in peripheral blood that included healthy and diseased individuals have been performed. Genome-wide transcriptomics studies were performed either on complex mixtures of blood cells, i.e. peripheral blood mononuclear cells [4], [5], [6], [7] or on isolated sub-populations of immune cells [8], [9], [10], [11]. Biological interpretation of results obtained with cell mixtures is extremely difficult and may lead to erroneous conclusions. Similarly, artefacts in measured levels of gene expression can arise as a consequence of lasting *ex vivo* cell-isolation procedures, leaving relatively low number of older datasets for correct biological interpretations [12], [13], [14], [15]. Metabolomics was shown to have a huge potential in investigation of physiological state, diagnosing diseases and measuring responses to various treatments [16], [17], [18], [19], [20]. While in metabolomics the idea of multiplex markers is already implemented, the studies which integrate data from several omics platforms are still very rare [21].

There are numerous reports showing that age, gender, ethnicity, diet and stress influence the numbers and functionalities of different immune cells, levels of antibodies and concentrations of bioactive factors that can be detected in peripheral blood samples. For example, in general, women are more efficient in mounting strong immune responses to infection than men, but are at the same time also more prone than males to suffer from autoimmune diseases, such as multiple sclerosis, rheumatoid arthritis and systemic lupus erythematosus [22]. Ageing is a physiological process that declines the percentages and functions of various types of immune cells, differently affecting males and females [23], [24], [25]. Nutrition can also cause functional changes in certain immune processes. For example, nutrition-related disorders such as anorexia and bulimia, as well as overweight (BMI 25.0–29.9) and obesity (BMI>30) are able to affect cellular, as well as humoral immune responses [26].

For a reliable clinical evaluation of complex molecular signatures it is especially important to carefully examine the physiological variability of all markers. Here we present a pilot study on 16 healthy individuals, applying combination of transcriptomics, metabolomics and cell biology tools in order to evaluate physiological variability of different peripheral blood measurables. We have focused on populations of several lymphocytes subtypes which we considered to be the most relevant for monitoring the effectiveness of novel cell-based immunotherapies of cancer. For gene expression studies in particular, isolated CD4^+^ T and natural killer (NK) cells were chosen, as the first ones are crucial for the induction and regulation of antigen-specific cellular and humoral adaptive immune responses and the second ones having important “missing self”-based and antibody-dependant cytolytic functions within innate immunity. The variability of different markers was evaluated in relation to sex, age, body mass index (BMI) and a day-to-day variance. Special care was taken to assure the correct experimental setup and to synchronise sampling and sample processing for all technological platforms used thereby avoiding sampling-related artefacts.

## Methods

### Blood sampling from healthy volunteers

This study was approved by the National Ethics Committee (Document No. 149/05/08). Peripheral blood samples were drawn from 16 healthy individuals after obtaining their signed informed consents. The selected experimental group was age and gender equilibrated (text S1). BMI was calculated for each individual enrolled. The inclusion criterion was age (20–60 years). The exclusion criteria were the following: acute or chronic diseases, pregnancy, smoking and taking oral contraception or other drugs. Every volunteer was screened for the viral and bacterial infection markers of blood-transmittable diseases (syphilis, HIV, hepatitis B and hepatitis C). Fasting morning blood samples were collected from each participant between 7 am and 9 am, on three separate days within a period of one month. Each time 43 ml of peripheral blood was drawn into three different types of Vacutainer tubes (BD Biosciences). For that purpose three 8 ml CPT, containing sodium chloride solution, two 8 ml K_2_EDTA and one 3 ml EDTA tubes were used. Blood samples were then immediately processed for isolation of selected lymphocyte subsets and a subsequent flow cytometry analysis, as described later. A basic haemogram analysis was also performed. Individuals were marked with a letter P (P as proband) and consecutive numbers, i.e. from P1 to P16. As blood samples from the individual P11 did not pass the threshold of normal cell counts, another healthy volunteer (P17) was selected and included into the study group. Blood samples of each individual were additionally labelled in order to distinguish between the 3 different sampling times, e.g. P1_D1, P1_D2, P1_D3 (D as day).

### Metabolite profiling

For the metabolite profiling analysis, lipid and polar fractions were extracted from plasma samples and subjected to gas chromatography coupled with mass spectrometry (GC-MS) and liquid chromatography coupled to tandem mass spectrometry (LC-MS/MS) analysis, respectively. Prior they were analysed by GC-MS, the samples were sequentially derivatised, while for LC-MS/MS analysis a technology allowing high sensitivity multiple reactions monitoring was applied. For the assessment of catecholamines and steroids, separate sample preparations and MS analytical approaches were performed, proprietary to Metanomics Gmbh, which carried out all metabolomics procedures. A schematic overview of the metabolite profiling analysis procedures is presented within the Text S1. A reference sample was prepared from all 48 plasma samples for a relative quantification of data. Only those measured values that were above the reliable quantification threshold limit were treated quantitatively and the rest of them only qualitatively. Data that were considered as quantitative were normalized against the reference sample and log_10_-transformed.

### Lymphocyte subpopulation counting

The expression of cell antigens was measured on lymphocytes from fresh whole blood collected into EDTA Vacutainer tubes. One hundred µl of peripheral human blood were incubated with 20 µl of CD25 FITC, CD4 PerCP, CD69 FITC, CD16 PE, HLA-DR PE and CD8 PerCP (all BD Biosciences) for 20 min at room temperature in a dark. Erythrocytes were lysed by incubation for 10 min with BD FACSTM Lysing Solution (BD Biosciences). Samples were centrifuged for 5 min at 450 g and subsequently washed twice with a cold phosphate-buffered saline (PBS). Cell pellets were then resuspended in 1 ml PBS. A total of 10000 events were collected. At least 2000 viable cells were gated and analyzed in each test. Lymphocytes were characterised by FSC vs. SSC followed by gating on CD4, CD8 and CD16 expression. Cell acquisition was performed using a BD FACSCanto flow cytometer. FlowJo version 7.6.1 (TreeStar) was utilized to analyze the percentage of CD25hi CD4+, HLA-DR+CD8+ T cells, and CD69+CD16+ NK cells.

### Isolation of CD4^+^ T and NK cells

Peripheral blood from each nine 8 ml Vacutainer CPT tubes (3 tubes/individual/sampling day), was processed according to the manufacturer's instructions to obtain a layer of mononuclear cells (MNCs) above the gel. The resulting MNCs from each CPT tube were transferred into a new separate container, gently mixed with the PBS buffer and then centrifuged for 10 min at 300 g at room temperature. The supernatants were discarded and the remaining MNC pellets were re-suspended, each in a 200 µl of PBS, supplemented with 2% foetal bovine serum (FBS) and 1 mM EDTA and transferred to separate 5 ml tubes. The first of triplicate MNC suspensions, pertaining to the same individual, whose blood was taken on a defined sampling day, was subjected to positive selection of CD4^+^ T cells by using the EasySep® Human CD4 Positive Selection Kit (Stemcell Technologies). The remaining MNC suspension was used for positive selection of NK cells with the EasySep® Human CD56 Positive Selection Kit (Stemcell Technologies). Both cell selection protocols were carried out according to the manufacturer's instructions. The isolated cells were immediately frozen and kept at −80°C until total RNA extraction. Prior to that, a small fraction of cells was removed, trypan blue stained for their vitality and counted in a Bürker-Türk counting chamber using an inverted optical microscope. The whole procedure was performed in less than 1 hour to minimise the stress related effects in transcriptomics datasets.

### Isolation and quality control of total RNA

Total RNA was extracted from isolated CD4^+^ T and NK cells with the RNeasy Mini Kit (Qiagen) according to manufacturer's instructions. Genomic DNA was removed by adding DNase I (Invitrogen) and RNA samples were concentrated with the RNeasy miniElute Cleanup Kit (Qiagen), as prescribed by the manufacturer. The quality and quantity of each total RNA sample was assessed with a NanoDrop ND-1000 spectrophotometer (NanoDrop Technologies) and the Agilent 2100 Bioanalyzer (Agilent Technologies).

### Microarray hybridizations and data preprocessing

The isolated RNA samples were first labelled with Illumina TotalPrep RNA Amplification Kit (Ambion) and subsequently hybridized to HumanWG-6 v3 Expression BeadChip (Illumina). After scanning, image acquisition was carried out with the BeadStudio version 3.3.7 software (Illumina).

Data preprocessing was performed in a R statistical environment [27], using the *lumi* software package for data input, quality control and robust spline normalization on log_2_-transformed data. According to quality control, pair-wise MA plots, density plots of signal intensity, as well as box plots were analysed for each microarray, both on raw and normalised data. In order to reduce the extent of false positive results, unexpressed genes with a detection p-value above 0.01, were filtered out in a subsequent data preprocessing step. For probe annotation, *annotate*, *lumiHumanAll.db* and *GO.db* Bioconductor software packages were used.

### Quantitative real-time PCR

TaqMan MGB™ dual-labelled probes (AssayOnDemand, Applied Biosystems) were used for real-time PCR analysis of the following selected genes: gamma 1 actin (ACTG1), adenylate cyclase-associated protein 1 (CAP1), coiled-coil-helix-coiled-coil-helix domain containing 2 (CHCHD2), glutathione S-transferase theta 1 (GSTT1), eukaryotic translation initiation factor 1A, Y-linked (EIF1AY), GTPase IMAP family member 7 (GIMAP7), granulysin (GNLY), interleukin 7 receptor (IL7R), DR beta 1 major histocompatibility complex, class II (HLA-DRB1) and DO beta major histocompatibility complex, class II (HLA-DOB). Two standard endogenous reference controls were also included, i.e. actin beta (ACTB) and 18S ribosomal RNA gene (18S). The cDNA synthesis, the qPCR reactions and data analysis were performed as described previously [28].

### Antibody concentrations

Antibody concentrations were determined with the Human IgG and IgM ELISA Quantification Set (Bethyl Laboratories) in 96 well high binding plates (Costar). Plasma samples were diluted with a blocking buffer as follows: 1∶1000 for IgM and 1∶10000 for IgG determination. The ABTS Single Solution (Zymed) was used for detection.

### Statistical analyses

All datasets were first visualised by principal component (PCA) and hierarchical clustering analysis (HCA) to overview the sample variability, using Multiexperiment Viewer software [29] (MeV).

In the metabolomic dataset a mixed model analysis of variance with biological factors, i.e. gender, BMI and age, up to the second order of interactions was applied. The sample age and time of sampling were considered as additional factors. Sample age, time of sampling, BMI and volunteer age were coded as numerical predictors and the gender as categorical one. Random effects were included in the model to take into account the variability of specific metabolite levels between individuals (SDsubject) and to compare them to technical and intra-individual variability (SDresidual). The ontology enrichment analysis was performed using Fisher's exact test.

Statistical testing (one-way ANOVA or t-test) of gender, age and BMI influences on the transcriptomics, flow cytometry and antibody concentrations datasets was performed using MeV [29]. Testing groups were defined to take into account the known physiological data and to ensure correct statistical analysis. For the latter, balanced number of samples in compared groups were used. Accordingly, 3 groups were formulated to assess the influence of age (<30, 30≥age≤50 and >50) and 3 for testing the BMI-related effects (lean: BMI<23, normal: 23≥BMI≤27, overweight: BMI>27) thereby creating equally represented clusters for statistical comparisons. Differentially expressed transcripts (p<0.01, logFC(abs)>0.85, FC – fold change) were functionally analysed according to Gene Ontology (GO), Kyoto Encyclopaedia of Genes and Genomes Onthology (KEGG) Pathways, Reactome Pathways and chromosome localization within the SystherDB toolbox [30], using Fisher's exact test. False discovery rate (FDR) multiple testing correction of p-values was applied where indicated [31]. To assess the physiological variability of gene expression within our group of healthy individuals, coefficients of variation (CV) in expression values were calculated over all analysed samples and R statistical environment [27]. Separately, the variability in gene expression was also estimated in samples collected from the same individual on three different days.

## Results

**Figure 1:**
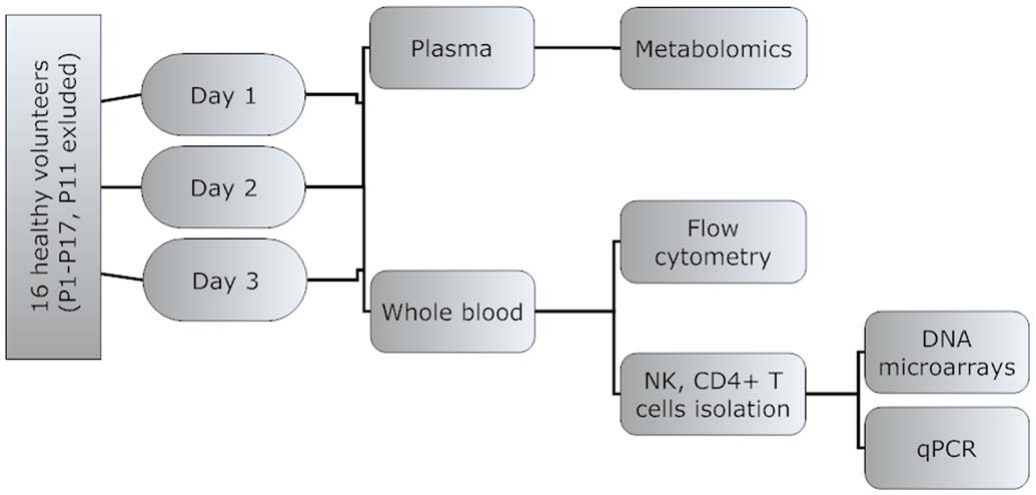
Overview of the experimental design. Sixteen gender and age matched healthy individuals were enrolled in a study. Fasting morning blood samples were taken on three days within one month and analysed using different omics approaches. Fasting morning samples were collected from 16 volunteer three times within a one month period.

Fasting morning samples of sixteen healthy volunteers were analysed in a “crossomics” study of the physiological variability. Blood samples from each volunteer were taken on three different days during a month period to assess the variability of markers in a single individual. Measurements of metabolites, concentrations of IgG and IgM antibodies, lymphocyte subset counts and gene expression in two selected immune cell types, namely CD4^+^ T cells and NK cells, were thus assessed in altogether 48 samples representing healthy population (Fig. 1).

### Metabolite profiles in blood plasma samples of healthy volunteers

Altogether 243 metabolites were identified and quantified from the collected plasma samples, 160 of them with a known structure and 83 still pending for their final structural identification. The acquired metabolite profiling data were analysed on two levels. A multivariate data overview was generated using principle component analysis (PCA) and hierarchical clustering analysis (HCA). Further, the effects of gender, age and BMI on metabolic profile of individuals were evaluated using statistical testing.

**Figure 2:**
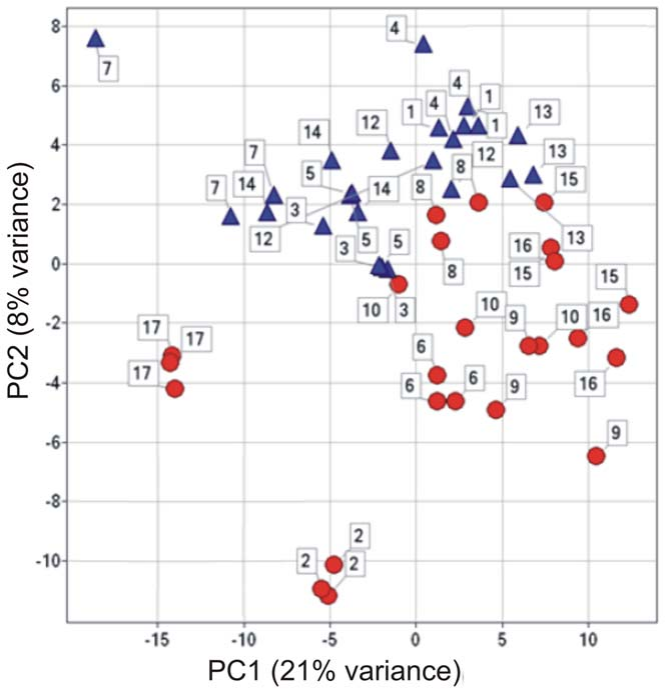
Overview of metabolite profiles variability found in plasma samples of healthy volunteers by principle component analysis score plot. Triangle – male volunteer, circle – female volunteer, coloured according to volunteers, labels shows sampling number within one volunteer. PC - principal component.

The PCA revealed high similarity of samples collected from the same individual on different days (Fig. 2). Using combination of PC1 and PC2 male and female samples separated, which indicates a strong impact of gender on metabolite profiles. These observations were confirmed also by the HCA (Text S1).

Mixed-effects model analysis of variance was performed to acquire the information on effects of gender, age and BMI on the metabolite profiles in collected samples. Gender was found to have the strongest effect on plasma metabolite profiles, followed by BMI and finally the age (Table 1, Table S1). The ontology enrichment analysis showed that statistically significant gender-attributed differences are mainly in amino acids, phospholipids and steroid hormones concentrations (Table 1). The highest difference in metabolite levels was detected for testosterone, being 14-fold higher in males than in females. Significantly higher values in males compared to females were also found for testosterone-17-sulfate, androstenedione and adrenaline. Similarly, levels of 9 amino acids (methionine, cysteine, isoleucine, leucine, proline, ketoleucine and indole-3-lactic acid) and several lipids (eicosapentaenoic acid, lysophosphatidylcholines C18:2 and C18:1) were found to be significantly higher in males, with more than 20% differences in their concentrations detected.

**Table 1:** The ontology enrichment analysis of metabolites that were identified as gender, age and BMI related or were identified as highly variable (HV, CV>0.5) in the analysed healthy population subset.

Ontology	ALL	Gender	BMI	Age	Variability
Amino acids and related	32	0.00 (24)	ns (2)	ns (5)	ns (2)
Phospholipids	20	ns (6)	ns (6)	0.03 (6)	ns (0)
Fatty acids	20	0.02 (1)	ns (2)	ns (1)	0.00 (9)
Cholesterol, bile acids, fatty alcohols and related	7	ns (0)	ns (1)	ns (0)	0.02 (4)
Steroids and related	8	0.03 (5)	ns (3)	ns (2)	0.00 (6)
**Total no. of DP/HV metabolites**	P<0.05	**35**	**33**	**29**	**39**
:male			45	30	
:female			20	21	

Total number of differentially present (DP) metabolites (p<0.05) related on gender, age, BMI, BMI within male individuals, age within male volunteers (:male), BMI within female volunteers and age within female volunteers (:female) are also given. Fisher's exact test was used to calculate significance of ontology enrichment (p-value is reported; ns – not significant). ALL - all metabolites within the ontology group. Number of differentially present metabolites in each category is given in brackets within each category.

**Figure 3:**
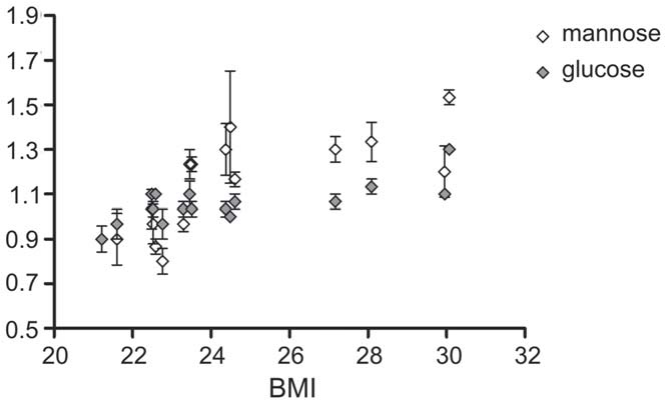
Average concentrations of two soluble sugars, glucose and mannose, are significantly increased in plasma samples of healthy volunteers with higher BMI values. p_Glucose_ = 0.0014, p_mannose_ = 0.0001. Error bars represent standard error within monthly measurements.

The levels of hormones were also found to be dependent on age and BMI, especially the ones belonging to the group of steroids and catecholamines (Table 1, Table S1). For example, androstenedione was decreasing with both age and BMI (Text S1). The most prominent group of metabolites detected through the ontology enrichment analysis that increased both with age and BMI, were however the lipids. Only phospholipids were found to be significantly regulated with age, app. 20% higher measured concentrations of lysophosphatidylcholine 16∶0 and phosphatidylcholine in older volunteers. Higher levels of sphingolipids, cholesterol, some fatty acids and glycolipids are also associated with higher BMI. In addition the increase in two soluble sugars, glucose and mannose, was associated with higher BMI values (Fig. 3).

In parallel, reliable estimates of residual, i.e. technical and intra-individual variability (SDresidual), as well as inter-individual standard deviations (SDsubject), were determined for 218 out of 243 metabolites. On average, SDsubject and SDresidual values were of similar size. However, few individual metabolites, metanephrine, normetanephrine, 3,4-dihydroxyphenylalanine (DOPA), pregnenolone sulphate, elaidic acid and proline, showed higher SDresidual in comparison to SDsubject values.

Variability of metabolites in healthy population was further evaluated by comparing coefficients of variability (CVs). Forty-one metabolites were identified with CV higher than 50% and 95 with CV higher than 30%. In ontology enrichment analysis, beside a set of lipids like cholesterol and different fatty acids, steroid hormones and some catecholamines, were additionally identified to be highly variable. Different xenobiotics, like coenzyme-Q, α-tocopherol, salicylic acid, caffeine, hippuric acid and others related to specific diet intake were also found to be variable (table 1).

### Fluctuations in lymphocyte subpopulations and antibody levels

Besides the numbers of total leucocytes, lymphocytes, NK cells (CD16^+^), cytotoxic CD8^+^ and CD4^+^ T cells that were determined in collected samples, the counts of activated forms of the same cell types (CD16^+^CD69^+^, CD8^+^HLA-DR^+^ and CD4^+^CD25^+^) were assessed by flow cytometry. In parallel, levels of IgG and IgM antibodies were measured in blood plasma samples.

**Figure 4:**
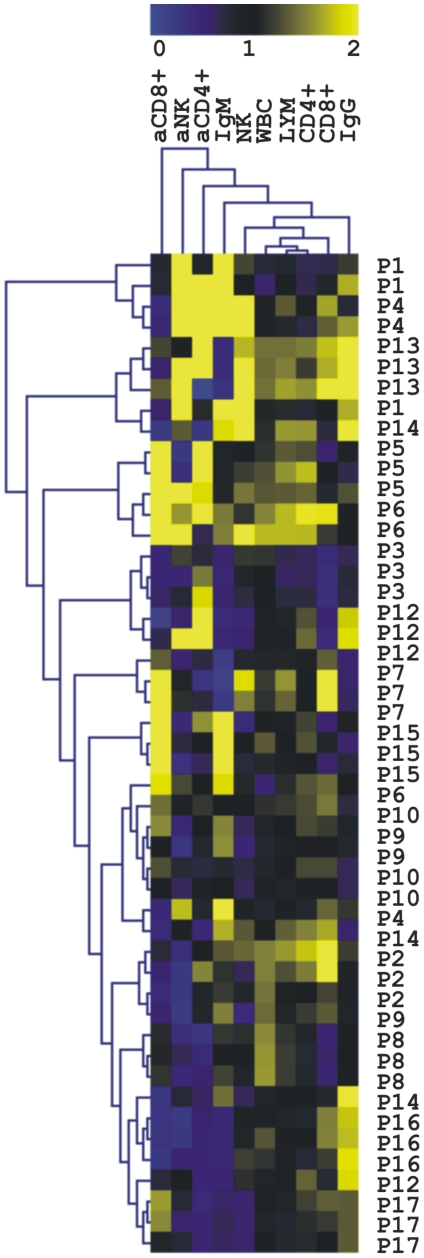
Variability of different lymphocyte subsets cell counts and antibody concentrations assessed in blood samples of healthy individuals. The HCA was performed both, for the analysis of samples and the variation of each parameter. The colour scale denotes increasing cell counts/concentrations of the measured parameters, with both extremes being low (blue) and high (yellow). The total numbers of leukocytes (WBC), lymphocytes (LYM), natural killer cells (NK), CD8^+^ and CD4^+^ T lymphocytes and their activated forms (aNK, aCD8^+^ T, aCD4^+^ T), were determined by flow cytometry. The concentrations of IgG and IgM antibodies are also included. Samples are marked by consecutive number of volunteer (P1–P17), volunteer gender (F, M) and sampling day (D1–5).

Similar as in metabolite profile analysis, the intra-individual variability was smaller than inter-individual one as shown by mulitivariate data analysis (Fig. 4). Statistical analysis of cell counts and antibody concentrations showed that the numbers of NK cells and their activated counterparts are gender-related (p_NK_ = 0.002, p_actNK_ = 0.001) with more than 50% higher cell counts determined in women. The count of activated CD4^+^ T cells were similarly more than 50% higher in women (p = 0.008). Interestingly, when studying the influence of BMI, only the CD4^+^ T cell counts were found to decrease significantly with increasing BMI (20% decrease, p = 4.6×10^−8^). In addition, the levels of CD8^+^ T subtype cell counts were found to decrease for 30% with age (p = 0.0003), while the percentage of activated CD8^+^ T cells increased two-fold in older individuals (p = 0.0004, Text S1, Table S2).

### Variability of gene expression in NK and CD4^+^ T cells

The transcriptomes of NK and CD4^+^ T cells were analysed separately, using whole genome microarrays (GEO Acc. No. GSE26163). We have focused on gene expression profiling of two selected lymphocyte subtypes instead of a more common and less tedious transcriptomics profiling of the whole lymphocyte population. The transcriptome profile of cell population depends both on the gene expression in each individual cell type as well as on the proportions of cell types and therefore contains biologically less relevant information compared to analysis of transcriptomes of separate cell types. When setting up the experimental design, special attention was given to sampling and sample processing in terms of minimizing the duration and standardizing the sample processing. All peripheral blood samples were processed in less than 1 h following their collection as it has been reported that within this time frame the sampling related stress response is not triggered yet [12], [32]. The variability in individual gene expression was analysed by multivariate methods (HCA, PCA) for both, the NK and CD4+ T cell data sets. The physiological variability of gene expression in healthy volunteers was assessed by considering CV as its measure.

**Figure 5:**
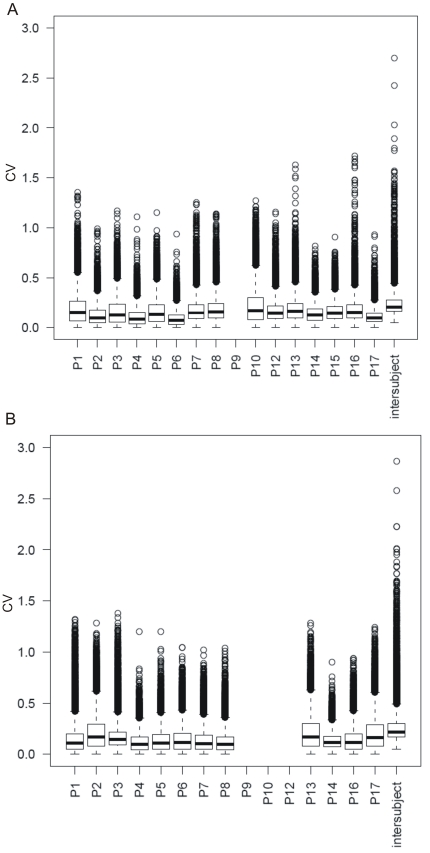
Intra-individual variability in gene expression compared to inter-individual variability. Variabilities are represented as boxplots of gene expression CV for each individual and in complete dataset. A) CD4^+^ T and B) NK cells. For subject P9 this evaluation was not possible for CD4^+^ T cell dataset as only one sample out of the three passed the technical quality control in all steps of the procedure. The same was true for subjects P9, P13 and P12 in NK cells dataset.

Altogether, 19746 transcripts were statistically significantly detected above background levels in NK and CD4^+^ T cell types. Similarly as in the case of metabolite profiling, the cell counting and antibody analysis, the multivariate overview showed that the intra-individual variability is smaller than the inter-individual one (Fig. 5, table 2). Some individual samples formed separate clusters in HCA (Text S1). These samples were compared to those, deviating from standard behaviour in case of IgG concentrations and percentages of both activated cell types. No sample overlapping was identified indicating that these differences were not due to the activation of each particular individual's immune system, at any of the sampling times.

**Table 2:** Percentage of transcripts with high and low variability was determined.

Cell type	High variability (CV>0.5)			Low variability
	Inter	Intra (avg)	Intra (max)	(CV<0.1)
**CD4^+^T**	4.1%	1.8%	4.5%	1.7%
**NK**	7.5%	2.3%	6.9%	2.2%

In parallel to the percentage of transcripts with high inter-individual variability (inter), percentage of transcripts with high variability within individual in monthly measurements were also calculated (intra); average percentage (intra(avg)) and maximum (intra(max) percentage of highly variable genes within 16 studied individuals are given. CV – coefficient of variability.

The percentage of highly variable transcripts was in both cell types below 10% (Table 2). Functional analysis of these transcripts showed that the most variable pathways are related to signalling (Text S1). Several immune related signalling pathways were identified as variable both in CD4^+^ T and NK cells, like Toll Cell Receptor related signalling, generation of second messenger molecules, cytokine signalling. Other interesting processes identified to be highly variable between healthy individuals were processes related to platelet aggregation and apoptosis. On the other hand, several pathways identified as highly stable are in both cell types related to transcription and translation, metabolism of glucose and energy metabolism.

**Figure 6:**
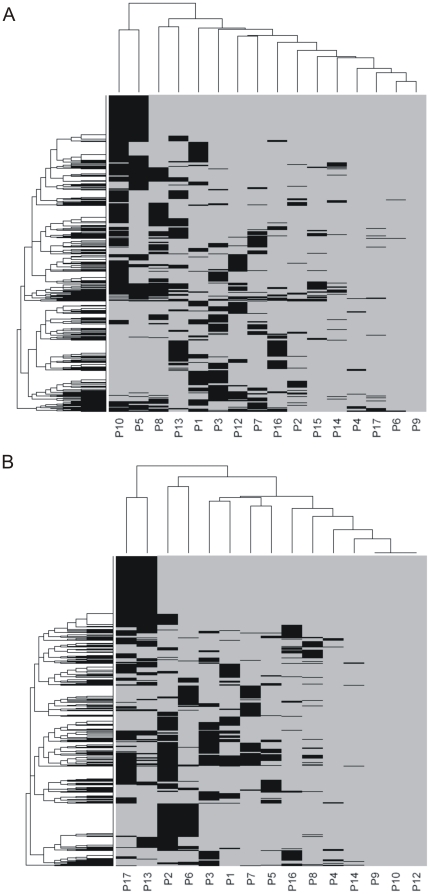
The pattern of transcripts with variable day-to-day expression seems to be in majority individuum-specific. Distribution of transcripts with high day to day variability in expression among healthy individuals for A) CD4^+^ T, and B) NK cells is shown. Transcripts identified as highly variable (CV>0.5) in at least one individual are ordered in rows of the heat map (1952 transcripts for CD4^+^ T and 2635 transcripts for NK cells). Individuals included in our study are listed in columns. Grey colour – the transcript was not identified as highly variable in the particular volunteer. Black colour – the transcript was identified as highly variable in particular volunteer.

Further, the gene expression variability within each of the volunteers during the sampling interval was evaluated (Fig. 5). Overall, all individuals had similar distribution of variability in gene expression, no major discrepancies were detected. One point to be noted however is that intra-individual variability (median CV app 0.1) is much lower than total variability in gene expression (median CV app. 0.2) as was also indicated by multivariate testing. Additionally, we checked if the same genes were identified as variable in different individuals. The pattern of genes with variable day-to-day expression (CV>0.5) seems to be in majority individuum-specific (Fig. 6).

### Physiological impacts on genes expression in CD4^+^ T and NK cells

**Figure 7:**
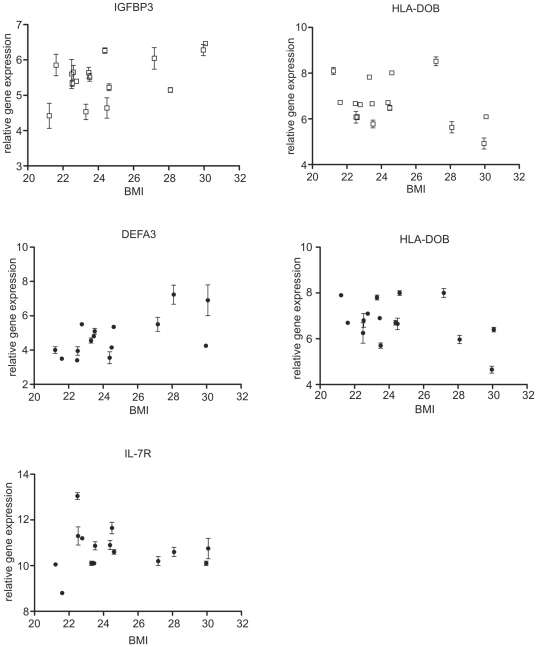
The most prominent BMI-related changes in gene expression were found for interleukin 7 receptor (IL7R), insulin-like growth factor binding protein 3 (IGFBP3), defensin 3 (DEFA3) and DO beta major histocompatibility complex, class II (HLA-DOB). CD4^+^ T cells - open squares, NK cells - closed circles. Error bars represent intra-individual standard error.

Statistical models to test and assess the effects of gender, age and BMI on the gene expression profile were applied. Overall, remarkably low number of differentially expressed (DE) transcripts was identified (Table 3). Individual inspection of DE transcripts showed that those with gender-related expression were located on x and y chromosomes, except GPR128, coding for the G-protein coupled receptor, which is located on chromosome 12. A non-protein coding X (inactive)-specific transcript (XIST) was the only gene with stronger expression in female volunteers, both in CD4^+^ T and NK cells. Several interleukine receptor genes, most prominently IL23R and IL7R, were found to be down-regulated with increasing BMI in NK cells (Fig. 7). The opposite was true for two defensin genes (DEFA1, DEFA3) which were both up-regulated with increased BMI in NK cells, with only DEFA3 being increased also in CD4^+^ T cells. In CD4^+^ T cells strong down-regulation of HLA-DOB gene, coding for the particular human leukocyte antigen (HLA) class II beta chain paralogue, was also detected with the increased BMI (Fig. 7). GPR128, insulin-like growth factor binding protein 3 (IGFBP3) and urotensin 2 (UTS2) genes were up-regulated in CD4^+^ T cells with the increasing age. The G-protein coupled receptor GPR128 was identified as DE as it was expressed only in one out of three samples in two individuals in NK cells (P10_D5, P12_D3) and only in one individual in CD4^+^ T cells (samples P12_D3, P12_D4, P12_D5). No particular characteristics of these samples were identified in other datasets collected, e.g. in either lymphocyte cell counts, immunoglobulin concentrations, metabolite or gene expression profile.

**Table 3:** Number of genes with sex, age or BMI-related expression in CD4^+^ T and NK cells.

	CD4^+^ T cells	NK cells
**Gender**	38 (17)	111 (17)
**Age (<30 versus >50)**	158 (9)	89 (5)
**BMI (<25 versus >27)**	73 (3)	1907 (52)

The cutoff for statistical significance was p<0.01. Numbers of differentially expressed (DE) genes with p<0.01 and relative log2 expression ratio between −0.85 and 0.85 is given in brackets. The overrepresentation analysis identified DE genes in the GO, KEGG and Reactome pathway ontologies did not produce any significant results.

### Verification of microarray results by real-time PCR

Genes identified to have either very low expression variability in both, CD4^+^ T and NK cells (ACTG1, CAP1, CHCHD2), high expression variability in both cell types (HLA-DRB1, GSTT1, EIF1AY) or high expression variability exclusively in CD4^+^ T (GIMAP7) or NK cells (GNLY), were chosen for the confirmatory qPCR analysis. In addition, two BMI-related genes, the first expressed in NK (IL7R) and the second in CD4^+^ T cells (HLA-DOB), were also included. Expression profile of selected genes determined by qPCR showed the same patterns as determined by DNA microarray hybridisations (supplemental data).

## Discussion

Novel immunotherapy approaches aiming especially at the treatment of cancer are becoming more and more promising. As there are numerous ongoing clinical studies and those still to be initiated which are testing the safety and effectiveness of such advanced therapies, there is a need for relevant and reliable control baseline values of different biological markers. The use of such controls would enable revealing of highly predictable, stable, robust and easy accessible diagnostic/prognostic markers.

Our study indicates that numerous blood measurable markers are remarkably stable. Within our healthy population sample, app. 20% of plasma metabolites and below 10% of genes expressed in NK and CD4^+^ cells respectively, had high variability in measured levels and would therefore most probably not be appropriate as clinically applicable diagnostic/prognostic markers. However, some of these variable determinants could still have some diagnostic or prognostic potential, especially if used in a combination with other markers, thereby forming a more complex and predictable molecular signature. The extent of intra-individual variability was found to be on average about half of the inter-individual one on all investigated levels, indicating the advantages for the so called personalised medicine strategies. However, certain metabolic substances, such as sex hormones catecholamines and glucocorticoids as well as some lipids, had higher intra-individual variability compared to the inter-individual one. This phenomenon can be attributed either to specific individual diet intake on or close to a particular sampling day and to highly variable endogenous, prevalently hormonal anabolism and catabolism in each individual, also reflecting his/her particular reactions to physical and psychical stressors. Expectedly, different xenobiotics, prevalently related to specific diet intake, were also characterised as highly variable.

While age, gender and ethnical influences are practically always accounted for in similar studies, other factors, like BMI and stress, being powerful players, able to harness the homeostasis of the human organism, are often not. Obese individuals experience chronic low-grade systemic immune activation accompanied by inflammation, which can be attributed to interactions between adipose tissue and immunocompetent cells [33]. Consequently, in obese individuals, increased incidence and severity of certain infectious diseases and occurrence of poor antibody responses to antigens, as compared to lean individuals, are observed [26], [34]. One however needs to note that the majority of studies analysing effects of obesity on immune responsiveness have been performed on morbidly obese individuals with BMI>50, while in our study, only four healthy individuals with BMI values between 27 to 30, were evaluated. The levels of certain cytokines, especially IL-6 and TNF-α, were found to be increased in subjects with high BMI values [35]. We did not measure levels of specific cytokines, however, we detected the decrease in transcription of two cytokine receptor genes, namely IL7R and IL23R in NK cells of healthy but overweight/obese volunteers (Fig. 7, supplemental data). This might be an interesting aspect to explore in studying effects of obesity. IL7R is involved in enhancement of NK cell maturation and survival [36] and is genetically associated with susceptibility to autoimmune diseases, especially multiple sclerosis [37]. IL23R has an impact on the activity of NK and especially Th17 cells [38] and may participate in acute response to infection in peripheral tissues as well as in certain autoimmune diseases, like morbus Chron [39]. Our finding that HLA-DOB gene is strongly down-regulated in CD4^+^ T cells with the increasing BMI is interesting and should be further explored, as scarce literature data is available regarding the presence of HLA-DO in T lymphocytes, showing that only weak DOB mRNA transcript expression can be detected in these cells [40]. Namely, HLA-DO molecules are expressed intracellularly, within the pre-lysosomal and lysosomal HLA-DR-containing compartments, prevalently in B lymphocytes and dendritic cells [40]. On the other hand defensin genes were found to be induced in individuals with higher BMI which is in agreement with the hypothesis that obese individuals experience continuous low grade systemic immune activation [33]. As one would expect, due to higher caloric input and a probable insulin resistance, the concentrations of certain lipids, glucose and mannose were also increased in individuals with higher BMI values (Fig. 3).

In general, our analysis reflects well-known effects of age as well as gender-related differences in basal anabolism and catabolism. Men have a higher muscle-to-fat ratio and they experience more extensive basal metabolic rate compared to women [41]. While sex- and stress-related hormones, some amino acids and most of the differentially present lipids were present in higher concentrations in males, those of phosphatidylcholine, creatine and taurine were found to be higher in females (Table 1), which is consistent with previous reports [42], [43], [44]. Expectedly, besides BMI, the age also influenced the levels of lipids and hormones, especially steroids and catecholamines (Table 1), as reported in previously published observations [45]. Gender- and age-related differences were also detected in cell counts of NK, CD8^+^ T lymphocytes and their activated counterparts. Higher activated NK and CD4^+^ T cell counts were found in women (supplemental data). In addition, the cell counts of both types of CD8^+^ T cells, but not CD4^+^ or NK cells were found to decrease with age (supplemental data), which is concordant with the existing data [46]. Interestingly however, almost no gender- or age-related differences were detected in gene expression profiles of CD4^+^ T and NK cells (supplemental data). Two diabetes related genes, coding for insulin-like growth factor (IGFBP3) and urotensin 2 (UTS2), were increasingly expressed with age which might indicate inclination of some individuals towards the development of type 2 diabetes. We are planning to monitor the health status of those in question and check if diabetes might evolve in the upcoming years.

This is the first “crossomic” study performed measuring variability of different components in peripheral blood of healthy Caucasoid individuals. Overall, the distribution of measured values in our dataset was in agreement with previously published data, providing that such information already existed, regardless of the fact that our sample of healthy individuals was rather small. The determined physiological baseline values for measured peripheral blood components can thus assist in development of effective diagnostics, prognostics and evaluation of responsiveness to various types of therapies by using complex molecular signatures biomarkers. Low intra-individual variability of different markers compared to the inter-individual one was measured on all investigated levels which indicates the importance of personalised medicine approaches from another perspective.

## Supporting Information

Text S1 **Overview of study experimental design, methodological approaches as well as some bioinformatics analysis that is less relevant or complementary to the ones in the main text of the manuscript.** QPCR results for verification of microarray analysis are also included.
(DOC)

Table S1 **Metabolic profiling approach. Datasheet with concentration ratios and results of ANOVA testing are given.**
(XLS)

Table S2 **Normalised data on lymophcyte counts and antibody concentration measurements, together with a detailed description of samples phenodata.** All abbreviations are used as explained in the text of main document.
(XLS)
