# Supplementary material for: A “Crossomics” Study Analysing Variability of Different Components in Peripheral Blood of Healthy Caucasoid Individuals
Source: PLoS One. 2012 Jan 12;7(1):e28761. doi: 10.1371/journal.pone.0028761 (PMC3257221; doi:10.1371/journal.pone.0028761)
Supplement: Text S1 — Overview of study experimental design, methodological approaches as well as some bioinformatics analysis that is less relevant or complementary to the ones in the main text of the manuscript. QPCR results for verification of microarray analysis are also included. (DOC) [file pone.0028761.s001.doc]

# Text S1 o the manuscript ‘A “crossomics” study analysing variability of different components in peripheral blood of healthy Caucasoid individuals’

1. **Overview of the experimental design.**

Sixteen gender and age matched healthy individuals were enrolled in a study. Fasting morning blood samples were taken on three days within one month and analysed using different omics approaches. As the isolation of CD4+ T and NK cells was not always technically successful, further samples were drawn on additional days from the same volunteer and they were marked accordingly (e.g. D4, ... up to D6).

**Correlation of BMI and age in individuals enrolled in the study.** The positive correlation between BMI and gender is significnat (Pearson correlation with p=0.006) but still regarded to be sufficiently small for simultaneous inclusion of both factors in the same ANOVA model (Pearson correlation: r=0.039). Triangle – males, circle – females.


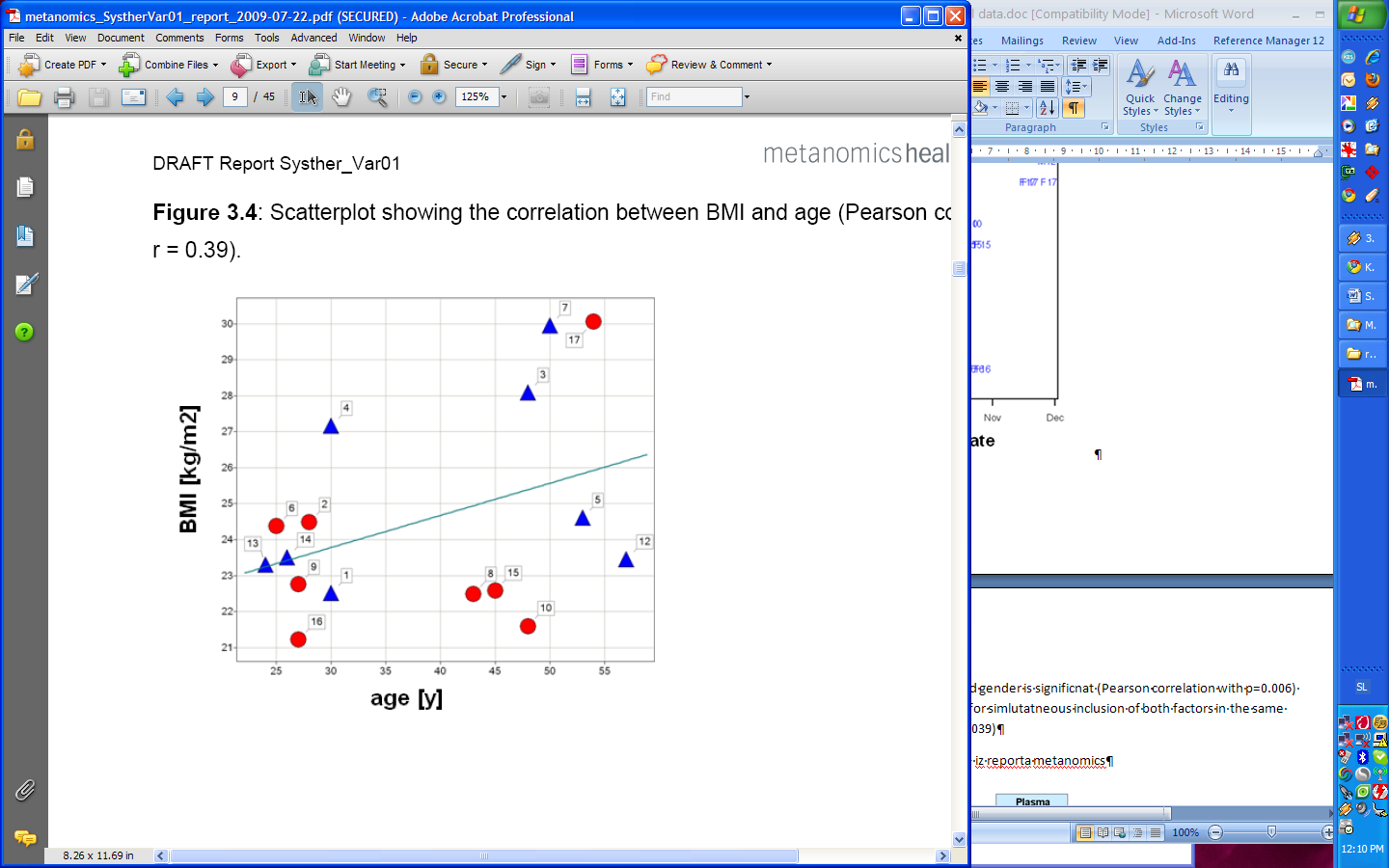


**2: Metabolomics of healthy volunteer plasma**

**Schematic overview of analytical approaches used for metabolite profiling.**


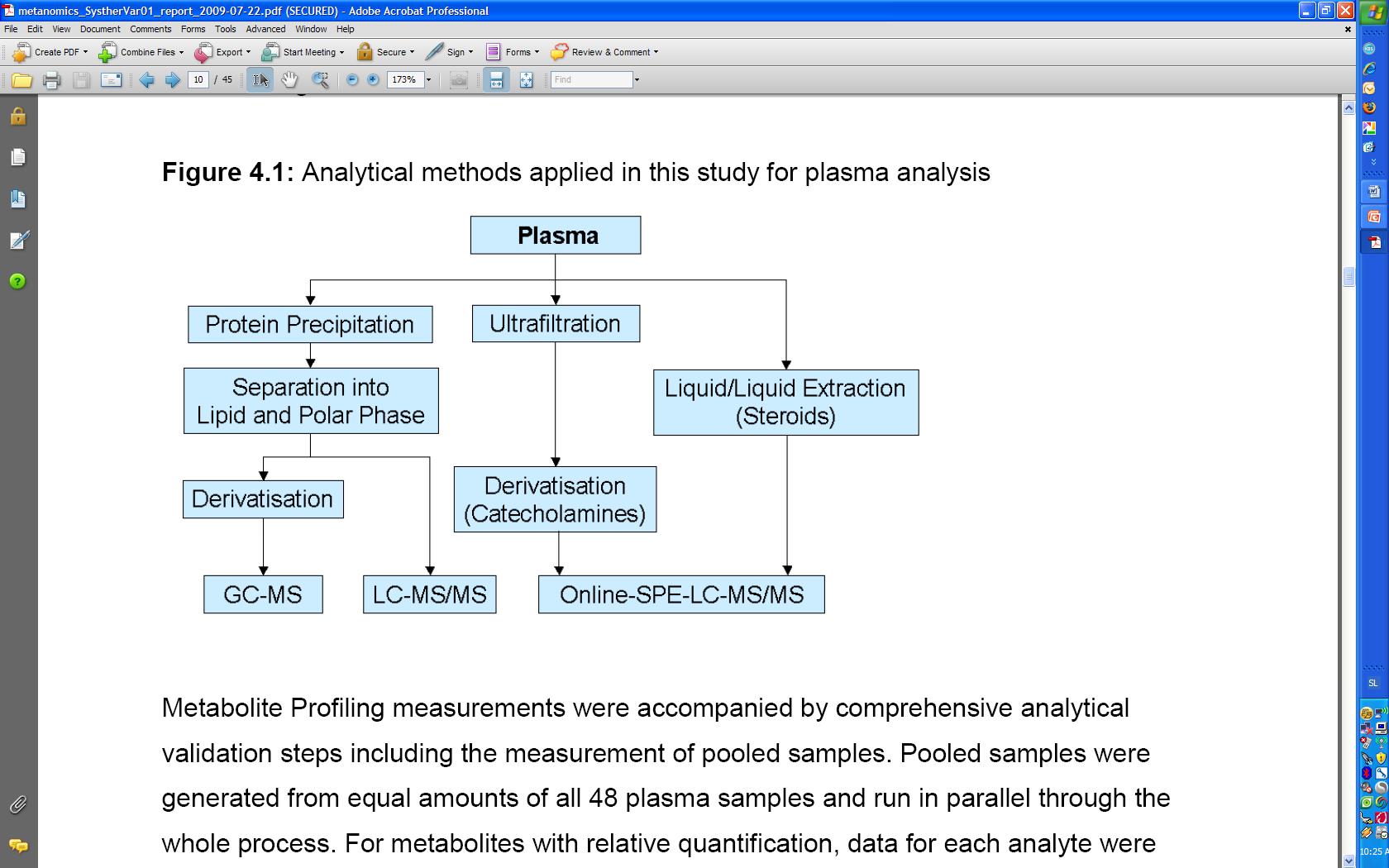


Legend: GC-MS – gas chromatography coupled with mass spectrometry, LC-MS/MS – liquid chromatography coupled to tandem mass spectrometry, multiple reaction monitoring profiling was applied. Online-SPE-LC-MS/MS – proprietary method of company Metanomics for highly specific and sensitive determinations of catecholamins and steroids (SPE – solid phase extraction).

An example of already described phenomena, which was detected also in our metabolic dataset:

Concentration of androstenedione, a common precursor of male and female sex hormones, significantly decreases with age (p = 0.04). Average values of three determinations per individual sample are presented. Error bars represent standard error within monthly measurements.


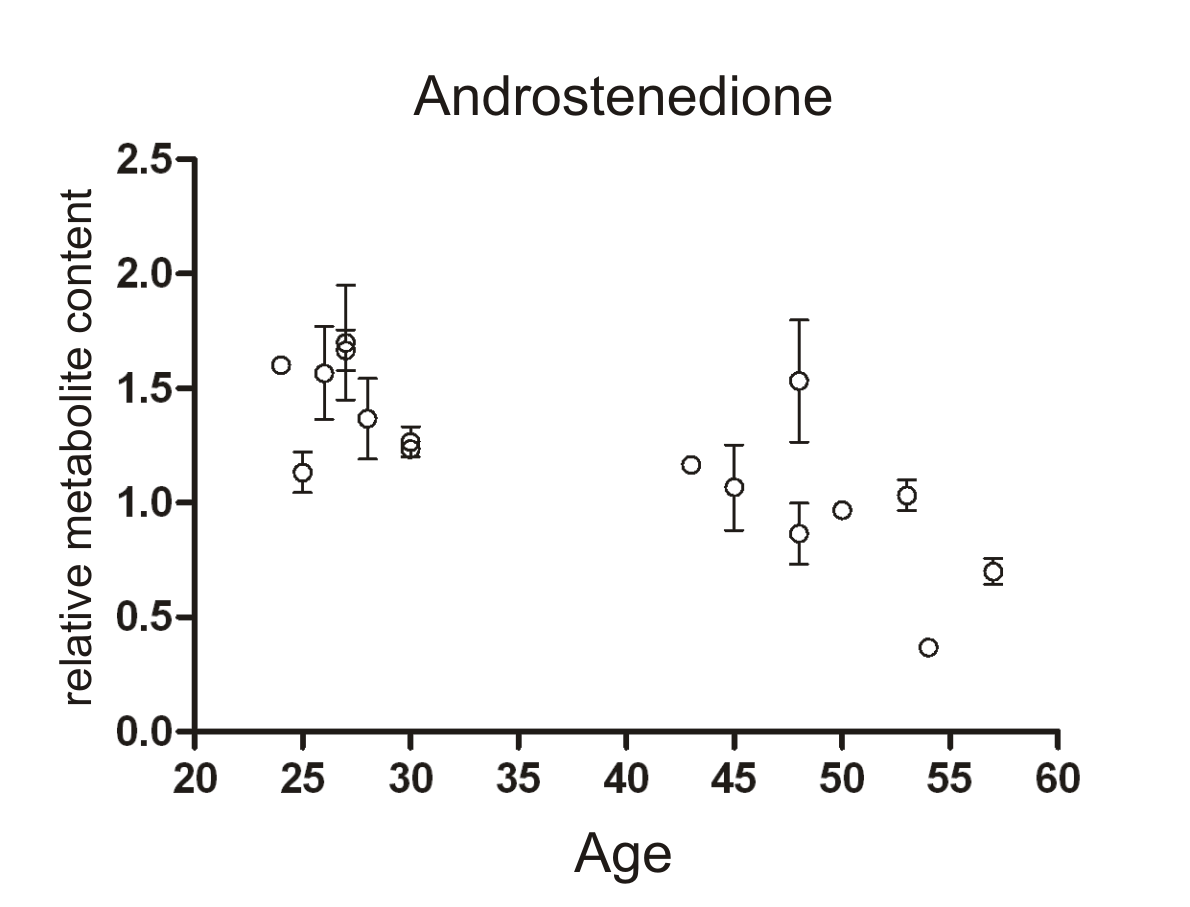


**Overview of metabolite profiles variability found in plasma samples of healthy volunteers by principle component analysis score plot.**

This figure complements analysis by principle component analysis (PCA) shown in Figure 2 of the manuscript.


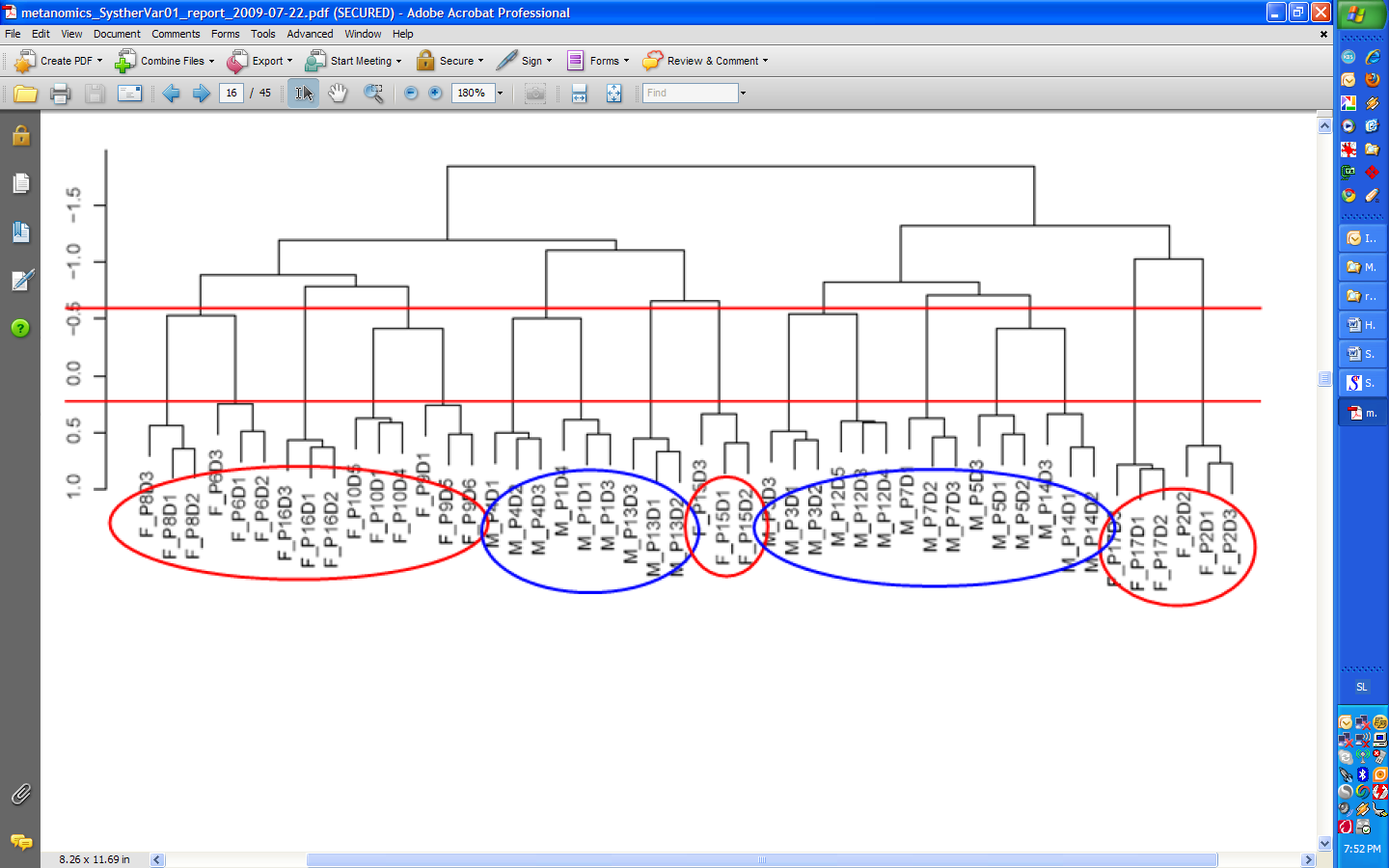


**3. Measurements of lymphocyte subset cell counts and immunoglobin concentrations**

**Day to day variance and inter-subject variance in lymphocyte subset populations and immunoglobulin concentrations.**

Similarity was evaluated using the Euclidian distance matrix. Darker red colour indicates higher similarity between the measured parameters. The largest intra-individual distance calculated was between 0.6 and 0.7 for volunteers P1, P4, P5 and P14. The inclusion of Ig concentration data increased the intra-individual distances, but didn’t have any effect on the general pattern of the distance matrix. The samples are signed with numbers from 1 to 48 (3 samples per each of 16 volunteers, for example 1 stands for P1D1, 2 for P1D2,...).


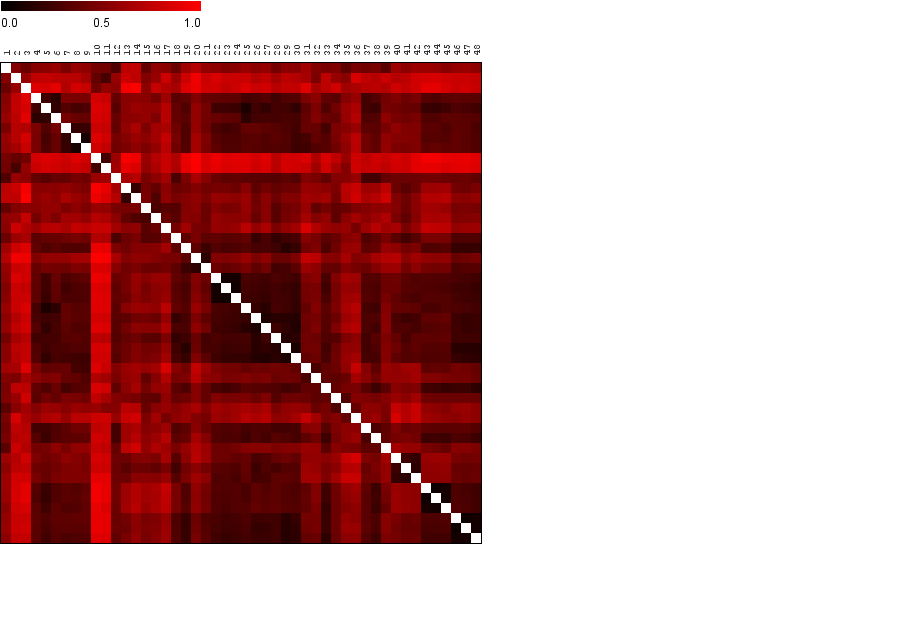


Number of CD8^+^ T cells is significantly decreasing with age both in male (closed diamond) and female (open diamond). FDR corrected p = 0.003. The average counts of monthly measurements are shown; error bars represent intra-individual standard error.


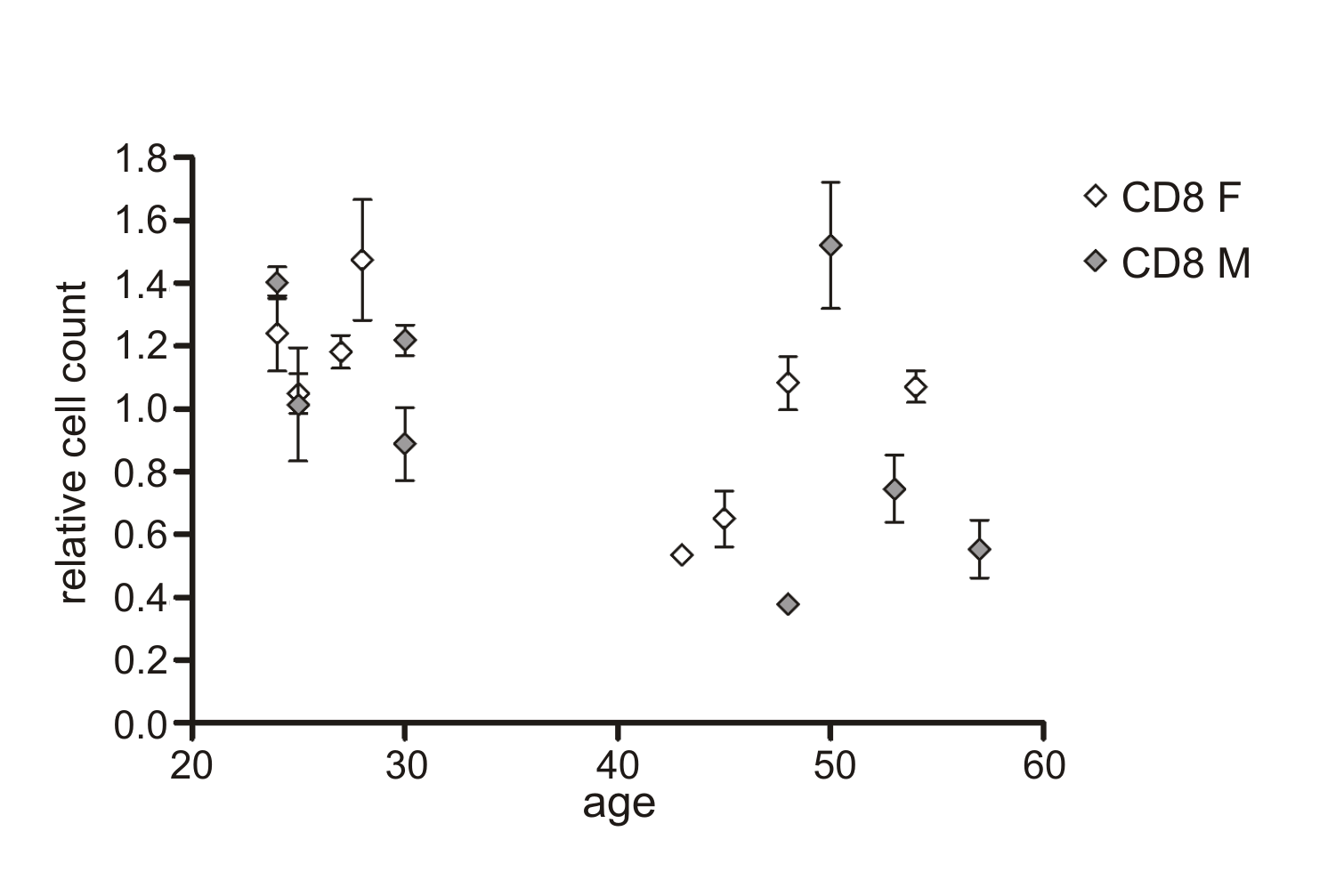


1. **Transcriptomics of CD4^+^ T and NK cells in healthy individuals**

**Analysis of DNA-microarray gene expression data**.

Raw datasets are deposited in GEO database under accession number GSE26163.

**Overview of lymphocyte gene expression profiles variability in healthy volunteers.**

The results of HCA for A) CD4^+^ T and B) NK cells are presented. Individuals are marked with letter P and a corresponding number; D1, D2 and D3 are marking the blood samples taken on different days during a period of 1 month.

A


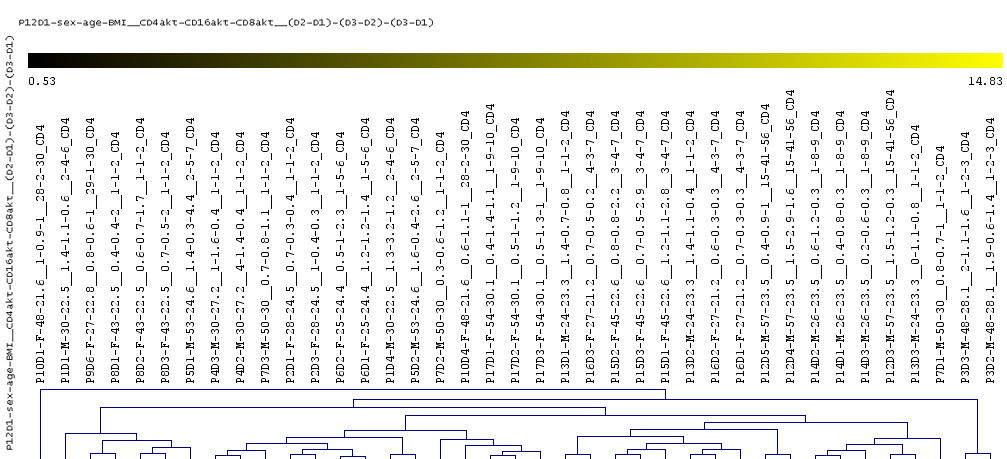


B


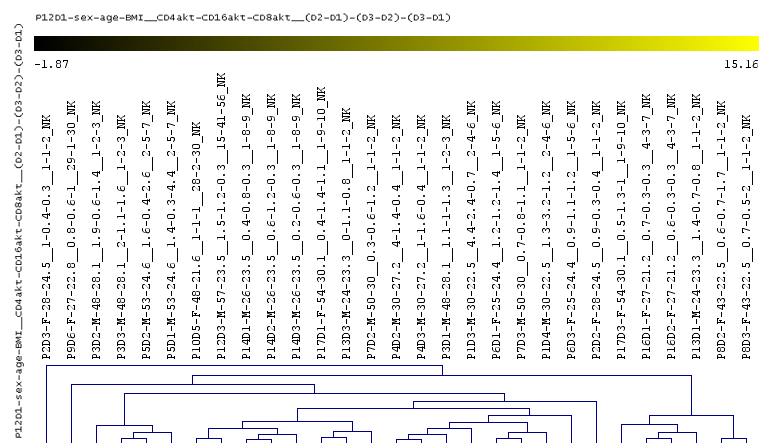


**Variability in measured gene expression data is not related to technical limitations of microarray technology.**

The general variability of gene expression in our volunteer group was calculated for all transcripts. Normalised gene expression values (M) were used for calculation of coefficients of variation (CV) in A) CD4^+^  T, and B) NK cells. Distribution of gene expression variability related to gene expression intensity in the healthy volunteers’ dataset was inspected. Higher variability is not correlated with relative expression values, which indicates its biological relevance and not the technical limits of the methodology.

A B

**Functional analysis of highly variable genes.**

Overrespresentation analysis of Reactome pathways is shown for genes with CV>0.3 in A) CD4^+^ T and B) NK cells and for genes with CV<0.1 in C) CD4^+^ T and D) NK cells. Only pathways with the FDR corrected P-values< 0.01 (p-value), generated using Fisher’s exact test are listed; DE genes – number of differentially expressed genes annotated to the specific pathway; All genes – total number of genes annotated to the particular group.

A)

| **Reactome Pathway** | **p-value** | **DE genes** | **ALL genes** |
| --- | --- | --- | --- |
| Signaling in Immune system | 7.81E-19 | 83 | 315 |
| Hemostasis | 7.62E-15 | 63 | 233 |
| Formation of Platelet plug | 5.58E-08 | 33 | 118 |
| Platelet Activation | 7.90E-08 | 30 | 101 |
| TCR signaling | 6.65E-08 | 26 | 76 |
| Packaging Of Telomere Ends | 9.60E-08 | 15 | 21 |
| Cell surface interactions at the vascular wall | 9.51E-08 | 28 | 91 |
| Downstream TCR signaling | 4.28E-07 | 23 | 67 |
| Generation of second messenger molecules | 5.64E-07 | 19 | 46 |
| Translocation of ZAP-70 to Immunological synapse | 5.22E-07 | 18 | 41 |
| Phosphorylation of CD3 and TCR zeta chains | 9.22E-07 | 18 | 43 |
| Elevation of cytosolic Ca++ levels | 1.67E-05 | 20 | 67 |
| Platelet degranulation | 1.68E-05 | 19 | 61 |
| Telomere Maintenance | 1.60E-05 | 16 | 43 |
| Apoptosis | 3.01E-05 | 28 | 129 |
| Response to elevated platelet cytosolic Ca++ | 3.16E-05 | 19 | 65 |
| Toll Receptor Cascades | 3.69E-05 | 15 | 41 |
| Exocytosis of Alpha granule | 1.17E-04 | 17 | 59 |
| Rho GTPase cycle | 4.83E-04 | 24 | 122 |
| Signaling by Rho GTPases | 4.83E-04 | 24 | 122 |
| Gene Expression | 4.68E-04 | 52 | 385 |
| Toll Like Receptor 4 (TLR4) Cascade | 4.64E-04 | 10 | 23 |
| Chemokine receptors bind chemokines | 8.96E-04 | 13 | 44 |
| Activated TLR4 signalling | 1.35E-03 | 9 | 21 |
| Signaling by PDGF | 1.73E-03 | 15 | 62 |
| Platelet activation triggers | 4.49E-03 | 10 | 32 |
| Innate Immunity Signaling | 4.47E-03 | 19 | 101 |
| Basigin interactions | 5.50E-03 | 9 | 27 |
| MyD88 cascade | 4.90E-03 | 7 | 16 |
| Toll Like Receptor 10 (TLR10) Cascade | 4.90E-03 | 7 | 16 |
| Toll Like Receptor 2 Cascade | 4.90E-03 | 7 | 16 |
| Toll Like Receptor 5 (TLR5) Cascade | 4.90E-03 | 7 | 16 |
| Toll Like Receptor 7/8 (TLR7/8) Cascade | 4.90E-03 | 7 | 16 |
| Toll Like Receptor TLR1:TLR2 Cascade | 4.90E-03 | 7 | 16 |
| Toll Like Receptor TLR6:TLR2 Cascade | 4.90E-03 | 7 | 16 |
| Integrin cell surface interactions | 5.78E-03 | 16 | 81 |
| Signalling by NGF | 6.29E-03 | 26 | 172 |
| Toll Like Receptor 9 (TLR9) Cascade | 7.47E-03 | 7 | 18 |
| Metabolism of amino acids | 7.45E-03 | 25 | 167 |
| Activation, myristolyation of BID and translocation to mitochondria | 8.95E-03 | 4 | 4 |

B)

| **Reactome Pathway** | **p-value** | | **DE genes** | | **ALL genes** | |  |
| --- | --- | --- | --- | --- | --- | --- | --- |
| Signaling in Immune system | 3.09E-23 | | 102 | | 315 | |  |
| Hemostasis | 1.79E-16 | | 74 | | 233 | |  |
| TCR signaling | 1.89E-09 | | 32 | | 76 | |  |
| Generation of second messenger molecules | 2.87E-09 | | 25 | | 46 | |  |
| Formation of Platelet plug | 3.75E-09 | | 39 | | 118 | |  |
| Phosphorylation of CD3 and TCR zeta chains | 3.73E-09 | | 24 | | 43 | |  |
| Translocation of ZAP-70 to Immunological synapse | 7.54E-09 | | 23 | | 41 | |  |
| Platelet Activation | 7.15E-09 | | 35 | | 101 | |  |
| Cell surface interactions at the vascular wall | 7.11E-09 | | 33 | | 91 | |  |
| Downstream TCR signaling | 5.16E-08 | | 27 | | 67 | |  |
| Exocytosis of Alpha granule | 2.87E-07 | | 24 | | 59 | |  |
| Platelet degranulation | 4.70E-07 | | 24 | | 61 | |  |
| Elevation of cytosolic Ca++ levels | 5.11E-07 | | 25 | | 67 | |  |
| Packaging Of Telomere Ends | 5.07E-07 | | 15 | | 21 | |  |
| Response to elevated platelet cytosolic Ca++ | 1.03E-06 | | 24 | | 65 | |  |
| Toll Receptor Cascades | 2.20E-05 | | 17 | | 41 | |  |
| Chemokine receptors bind chemokines | 5.81E-05 | | 17 | | 44 | |  |
| Apoptosis | 1.11E-04 | | 30 | | 129 | |  |
| Signalling by NGF | 1.06E-04 | | 36 | | 172 | |  |
| Toll Like Receptor 4 (TLR4) Cascade | 1.10E-04 | | 12 | | 23 | |  |
| Activated TLR4 signalling | 2.63E-04 | | 11 | | 21 | |  |
| Immunoregulatory interactions between a Lymphoid and a non-Lymphoid cell | 3.25E-04 | | 27 | | 118 | |  |
| Nef-mediates down modulation of cell surface receptors by recruiting them to clathrin adapters | 3.24E-04 | | 11 | | 22 | |  |
| Telomere Maintenance | 3.52E-04 | | 15 | | 43 | |  |
| Signaling by PDGF | 5.57E-04 | | 18 | | 62 | |  |
| Opioid Signalling | 5.42E-04 | | 20 | | 75 | |  |
| The role of Nef in HIV-1 replication and disease pathogenesis | 5.89E-04 | | 12 | | 29 | |  |
| MyD88 cascade | 6.36E-04 | | 9 | | 16 | |  |
| Toll Like Receptor 10 (TLR10) Cascade | 6.36E-04 | | 9 | | 16 | |  |
| Toll Like Receptor 2 Cascade | 6.36E-04 | | 9 | | 16 | |  |
| Toll Like Receptor 5 (TLR5) Cascade | 6.36E-04 | | 9 | | 16 | |  |
| Toll Like Receptor 7/8 (TLR7/8) Cascade | 6.36E-04 | | 9 | | 16 | |  |
| Toll Like Receptor TLR1:TLR2 Cascade | 6.36E-04 | | 9 | | 16 | |  |
| Toll Like Receptor TLR6:TLR2 Cascade | 6.36E-04 | | 9 | | 16 | |  |
| M Phase | 7.53E-04 | | 21 | | 86 | |  |
| HIV Infection | 8.97E-04 | | 35 | | 192 | |  |
| Early Phase of HIV Life Cycle | 9.46E-04 | | 8 | | 13 | |  |
| Toll Like Receptor 9 (TLR9) Cascade | 1.23E-03 | | 9 | | 18 | |  |
| Cell Cycle, Mitotic | 1.20E-03 | | 47 | | 294 | |  |
| Death Receptor Signalling | 1.28E-03 | | 8 | | 14 | |  |
| Extrinsic Pathway for Apoptosis | 1.28E-03 | | 8 | | 14 | |  |
| Integrin cell surface interactions | 2.22E-03 | | 19 | | 81 | |  |
| Muscle contraction | 2.28E-03 | | 11 | | 31 | |  |
| Striated Muscle Contraction | 2.28E-03 | | 11 | | 31 | |  |
| Apoptotic execution phase | 2.32E-03 | | 13 | | 43 | |  |
| Receptor-ligand complexes bind G proteins | 4.51E-03 | | 28 | | 154 | |  |
| Gene Expression | 4.72E-03 | | 55 | | 385 | |  |
| Innate Immunity Signaling | 4.77E-03 | | 21 | | 101 | |  |
| Signaling by EGFR | 5.19E-03 | | 13 | | 47 | |  |
| Formation of Fibrin Clot (Clotting Cascade) | 5.18E-03 | | 10 | | 29 | |  |
| Host Interactions of HIV factors | 5.18E-03 | | 24 | | 126 | |  |
| Metabolism of proteins | 5.14E-03 | | 33 | | 199 | |  |
| Mitotic Prometaphase | 5.23E-03 | | 18 | | 82 | |  |
| Binding and entry of HIV virion | 5.97E-03 | | 5 | | 6 | |  |
| Platelet Adhesion to exposed collagen | 6.15E-03 | | 6 | | 10 | |  |
| Apoptotic cleavage of cellular proteins | 6.59E-03 | | 11 | | 37 | |  |
| Platelet activation triggers | 7.81E-03 | | 10 | | 32 | |  |
| Nef mediated downregulation of MHC class I complex cell surface expression | | 8.11E-03 | | 6 | | 11 | |
| Basigin interactions | 9.22E-03 | | 9 | | 27 | |  |
| Metabolism of amino acids | 9.22E-03 | | 28 | | 167 | |  |
| Formation of tubulin folding intermediates by CCT/TriC | 9.74E-03 | | 8 | | 22 | |  |

C)

| **Reactome Pathway** | **p-value** | **DE genes** | **ALL genes** |
| --- | --- | --- | --- |
| Influenza Viral RNA Transcription and Replication | 7.70E-12 | 19 | 150 |
| Influenza Life Cycle | 5.19E-12 | 19 | 154 |
| Influenza Infection | 5.61E-12 | 19 | 159 |
| Gene Expression | 3.61E-11 | 26 | 385 |
| Viral mRNA Translation | 1.95E-09 | 13 | 85 |
| Eukaryotic Translation Termination | 1.73E-09 | 13 | 87 |
| Peptide chain elongation | 1.73E-09 | 13 | 87 |
| Eukaryotic Translation Elongation | 2.40E-09 | 13 | 90 |
| Cap-dependent Translation Initiation | 3.68E-09 | 14 | 115 |
| Eukaryotic Translation Initiation | 3.68E-09 | 14 | 115 |
| Formation of a pool of free 40S subunits | 4.41E-09 | 13 | 97 |
| Metabolism of proteins | 5.43E-09 | 17 | 199 |
| Translation | 5.38E-09 | 14 | 122 |
| 3 -UTR-mediated translational regulation | 9.28E-09 | 13 | 107 |
| L13a-mediated translational silencing of Ceruloplasmin expression | 9.28E-09 | 13 | 107 |
| GTP hydrolysis and joining of the 60S ribosomal subunit | 1.14E-08 | 13 | 108 |
| Diabetes pathways | 1.11E-08 | 17 | 215 |
| Integration of energy metabolism | 4.72E-07 | 15 | 214 |
| Formation of the ternary complex, and subsequently, the 43S complex | 2.57E-06 | 8 | 51 |
| Glucose Regulation of Insulin Secretion | 2.45E-06 | 12 | 150 |
| Vif-mediated degradation of APOBEC3G | 2.84E-06 | 8 | 53 |
| Ribosomal scanning and start codon recognition | 5.59E-06 | 8 | 58 |
| Translation initiation complex formation | 5.59E-06 | 8 | 58 |
| Activation of the mRNA upon binding of the cap-binding complex and eIFs, and subsequent binding to 43S | 5.56E-06 | 8 | 59 |
| Autodegradation of Cdh1 by Cdh1:APC/C | 8.21E-06 | 8 | 63 |

D)

| **Reactome Pathway** | **p-value** | **DE genes** | **ALL genes** |
| --- | --- | --- | --- |
| Influenza Viral RNA Transcription and Replication | 7.70E-12 | 19 | 150 |
| Influenza Life Cycle | 5.19E-12 | 19 | 154 |
| Influenza Infection | 5.61E-12 | 19 | 159 |
| Gene Expression | 3.61E-11 | 26 | 385 |
| Viral mRNA Translation | 1.95E-09 | 13 | 85 |
| Eukaryotic Translation Termination | 1.73E-09 | 13 | 87 |
| Peptide chain elongation | 1.73E-09 | 13 | 87 |
| Eukaryotic Translation Elongation | 2.40E-09 | 13 | 90 |
| Cap-dependent Translation Initiation | 3.68E-09 | 14 | 115 |
| Eukaryotic Translation Initiation | 3.68E-09 | 14 | 115 |
| Formation of a pool of free 40S subunits | 4.41E-09 | 13 | 97 |
| Metabolism of proteins | 5.43E-09 | 17 | 199 |
| Translation | 5.38E-09 | 14 | 122 |
| 3 -UTR-mediated translational regulation | 9.28E-09 | 13 | 107 |
| L13a-mediated translational silencing of Ceruloplasmin expression | 9.28E-09 | 13 | 107 |
| GTP hydrolysis and joining of the 60S ribosomal subunit | 1.14E-08 | 13 | 108 |
| Diabetes pathways | 1.11E-08 | 17 | 215 |
| Integration of energy metabolism | 4.72E-07 | 15 | 214 |
| Formation of the ternary complex, and subsequently, the 43S complex | 2.57E-06 | 8 | 51 |
| Glucose Regulation of Insulin Secretion | 2.45E-06 | 12 | 150 |
| Vif-mediated degradation of APOBEC3G | 2.84E-06 | 8 | 53 |
| Ribosomal scanning and start codon recognition | 5.59E-06 | 8 | 58 |
| Translation initiation complex formation | 5.59E-06 | 8 | 58 |
| Activation of the mRNA upon binding of the cap-binding complex and eIFs, and subsequent binding to 43S | 5.56E-06 | 8 | 59 |
| Autodegradation of Cdh1 by Cdh1:APC/C | 8.21E-06 | 8 | 63 |

**Comparison of NK and CD4^+^ T cells gene expression profiles**

When comparing gene expressions between NK and CD4+ T cells, more DE transcripts were identified. Out of 1992 significant DE transcripts, 1094 DE transcripts with log2 expression ratio between -0.85 and 0.85 (Mabs>0.85) were detected. The overrepresentation analysis of genes with higher expression in both cell types was performed separately, using GO, KEGG Pathways, Reactome Pathways and chromosome localization ontologies.

Genes belonging to the pathways related to Toll-Like Receptor (TLR) signalling were found to be expressed stronger in CD4+ T cells. Some additional interesting pathways were also detected to be more active in CD4+ T cells as compared to NK cells, for example ZAP-70 immunological synapse translocation, myeloid differentiation primary response gene 88 (MyD88) cascade, Ca++ signalling, haemostasis, platelet activation and gene expression associated to regulation of translation. In NK cells, cytokine receptor genes and genes involved in regulatory interactions between lymphoid and non-lymphoid cells were significantly more active than in CD4+ T cells. This is once again in-line with the physiology and the known functions of NK cells. When performing the overrepresentation analysis using KEGG pathway database, NK cell mediated cytotoxicity was the most significantly regulated pathway (see table below).

**Functional analysis of gene expression differences between CD4^+^ T and NK cells.** Overrepresentation analysis of Reactome pathways is shown. Pathways representing genes with higher expression in A) CD4^+^ T, and B) NK cells are displayed. Only pathways with the false discovery rate corrected p-values < 0.01 (p-value), generated using Fisher’s exact test are listed; DE genes – number of differentially expressed genes annotated to the specific pathway; All genes – total number of genes annotated to the particular group. TCR - T cell receptor, MyD88 - myeloid differentiation primary response gene 88, TLR - Toll-Like Receptor, ZAP-70 - zeta chain (TCR) associated protein kinase 70kDa. The GO and KEGG pathway ontologies enrichment analysis resulted in functionally similar results.

| 1. **Reactome Pathway** | **DE genes** | **All genes** | **p-value** |
| --- | --- | --- | --- |
| Signaling in Immune system | 27 | 315 | 0.0000 |
| Phosphorylation of CD3 and TCR zeta chains | 10 | 43 | 0.0000 |
| TCR signaling | 12 | 76 | 0.0000 |
| Downstream TCR signaling | 11 | 67 | 0.0000 |
| Toll Receptor Cascades | 9 | 41 | 0.0000 |
| Toll Like Receptor 4 (TLR4) Cascade | 7 | 23 | 0.0000 |
| Toll Like Receptor 10 (TLR10) Cascade | 6 | 16 | 0.0000 |
| Toll Like Receptor 2 Cascade | 6 | 16 | 0.0000 |
| Toll Like Receptor 5 (TLR5) Cascade | 6 | 16 | 0.0000 |
| Toll Like Receptor 7/8 (TLR7/8) Cascade | 6 | 16 | 0.0000 |
| Toll Like Receptor TLR1:TLR2 Cascade | 6 | 16 | 0.0000 |
| Toll Like Receptor TLR6:TLR2 Cascade | 6 | 16 | 0.0000 |
| Toll Like Receptor 9 (TLR9) Cascade | 6 | 18 | 0.0000 |
| Activated TLR4 signalling | 6 | 21 | 0.0001 |
| MyD88 cascade | 6 | 16 | 0.0000 |
| Translocation of ZAP-70 to Immunological synapse | 9 | 41 | 0.0000 |
| Generation of second messenger molecules | 9 | 46 | 0.0000 |
| Hemostasis | 16 | 233 | 0.0001 |
| Innate Immunity Signaling | 10 | 101 | 0.0003 |
| Platelet Activation | 10 | 101 | 0.0003 |
| Elevation of cytosolic Ca++ levels | 8 | 67 | 0.0004 |
| Metabolism of carbohydrates | 9 | 94 | 0.0007 |
| Formation of Platelet plug | 10 | 118 | 0.0007 |
| Response to elevated platelet cytosolic Ca++ | 7 | 65 | 0.0020 |
| Translation | 9 | 122 | 0.0046 |
| Glucose metabolism | 7 | 77 | 0.0057 |
| 3 -UTR-mediated translational regulation | 8 | 107 | 0.0072 |
| L13a-mediated translational silencing of Ceruloplasmin expression | 8 | 107 | 0.0072 |
| Exocytosis of Alpha granule | 6 | 59 | 0.0070 |
| GTP hydrolysis and joining of the 60S ribosomal subunit | 8 | 108 | 0.0068 |
| Platelet degranulation | 6 | 61 | 0.0094 |

| 1. **Reactome Pathway** | **DE genes** | **All genes** | **p-value** |
| --- | --- | --- | --- |
| Immunoregulatory interactions between a Lymphoid and a non-Lymphoid cell | 19 | 118 | 0.0000 |
| Signaling in Immune system | 28 | 315 | 0.0000 |
| Hemostasis | 17 | 233 | 0.0000 |
| Platelet Activation | 10 | 101 | 0.0006 |
| Formation of Platelet plug | 10 | 118 | 0.0022 |
| Chemokine receptors bind chemokines | 6 | 44 | 0.0056 |

**Verification of microarray results by quantitative real-time PCR.**

Varability in gene expression as determined by microarray and qPCR approaches: CVs were calculated for expresssion values determined by both methodologies

| **NK** | **ACTG1** | **IL7R** | **GIMAP7** | **GSTT1** | **GNLY** | **CAP1** | **CHCHD2** |
| --- | --- | --- | --- | --- | --- | --- | --- |
| qPCR | 0.00 | 1.94 | 0.98 | 1.20 | 0.89 | 0.78 | 1.03 |
| Microarray | 0.09 | 0.96 | 0.16 | 1.03 | 0.20 | 0.09 | 0.07 |

| **CD4^+^ T** | **ACTB** | **ACTG1** | **CAP1** | **CHCHD2** | **HLA-DRB1** | **GIMAP7** | **GNLY** | **GSTT** | **EIF1AY** | **IL7R** | **HLA-DOB** |
| --- | --- | --- | --- | --- | --- | --- | --- | --- | --- | --- | --- |
| qPCR | 0.12 | 0.12 | 0.15 | 0.21 | 1.72 | 0.18 | 1.05 | 0.76 | 0.98 | 0.23 | 0.77 |
| Microarray | 0.10 | 0.08 | 0.08 | 0.07 | 1.23 | 0.15 | 1.34 | 0.70 | 0.95 | 0.23 | 0.75 |

BMI-related differences in gene expression as measured by qPCR

Statistical testing was performed for qPCR with the same groups setting as in microarray analysis.

| p-value | IL7R | HLA-DOB |
| --- | --- | --- |
| CD4+ T | 0.0351 | 0.0023 |
| NK | 0.0682 | NA |

NA-not applicable
